# Need for velopharyngeal surgery after primary palatoplasty in cleft patients. A retrospective cohort study and review of literature

**DOI:** 10.1016/j.amsu.2021.102707

**Published:** 2021-08-12

**Authors:** Ana Tache, Youri Maryn, Maurice Y. Mommaerts

**Affiliations:** a Cleft & Craniofacial Team, Universitair Ziekenhuis Brussel, Vrije Universiteit Brussel, Belgium; b Department of Otorhinolaryngology and Head & Neck Surgery, European Institute for ORL-HNS, GZA Sint-Augustinus, Wilrijk, Belgium

**Keywords:** Cleft palate, Velopharyngeal insufficiency, Pharyngoplasty, Speech

## Abstract

**Background:**

Enabling intelligible speech plays an important role in achieving social inclusion and a good quality of life of cleft patients. A crude measure of primary palatal repair quality is the incidence of operations to correct velopharyngeal insufficiency (VPI) after speech-language therapy has proven inadequate. This study assessed the necessity for surgery to correct velopharyngeal insufficiency following our standardized two-staged protocol, compared the results with the literature, and identified factors that may influence velopharyngeal competence.

**Methods:**

A review of the literature was performed. The outcome measure in our series was the necessity for a secondary procedure to correct velopharyngeal insufficiency. The results of literature review were compared with the results of our case series, which we treated using a standardized protocol.

**Results:**

In our retrospective study, 5 patients (2.5%) required secondary pharyngoplasty. In literature, the frequency of surgery to correct velopharyngeal insufficiency after one- and two-stage protocols were 13.6% and 24.5%, respectively. No statistical difference was found between bilateral and unilateral clefts. The frequencies of velopharyngeal surgery were 7.2% after Furlow palatoplasty, 17.5% after a 2-flap palatoplasty, 18.6% after a Wardill-Killner palatoplasty, and 35.6% after a Von Langenbeck palatoplasty.

**Conclusion:**

The literature reported that one-stage palatoplasty is correlated with a lower incidence of secondary pharyngeal surgery. Our standardized two-stage protocol proved successful in avoiding secondary velopharyngeal surgery but due to the reduced number of patients included in our study, more research is needed.

## Highlights

• A crude measure of primary palatal repair quality is the incidence of pharyngoplasty.
• Following our standardized two-stage protocol, 2.9% patients required secondary pharyngoplasty.
• In literature, the frequency of pharyngeal surgery after one-stage protocol was 13.6%.
• In literature, the frequency of pharyngeal surgery after a two-stage protocol was 24.5%.

## 1 Introduction

Important goals of treatment of cleft patients are their social inclusion and normal quality of life. Enabling intelligible speech plays an important role in achieving these. A crude measure of primary palatal repair quality is velopharyngeal insufficiency (VPI) and the incidence of operations to correct velopharyngeal insufficiency after speech-language therapy has proven inadequate.

A higher frequency of velopharyngeal surgery has been correlated with the technique and timing of palatal repair, gender, type of clefting and experience of the surgeon [1]. Over the years, several techniques and protocols have been developed to the with the aim of improving the speech outcomes, by optimizing the anatomy of the repaired cleft velopharynx. Currently there is lack of consensus as to which surgical technique yields optimal speech results and an important proportion of the patients require a second operation to correct velopharyngeal insufficiency.

This study assessed the necessity for surgery to correct VPI following our standardized two-staged protocol, compared the results with the current existing literature, and identified factors that may influence velopharyngeal competence.

## 2 Material and methods

### 2.1 Study design and participants

This retrospective observational cohort study has been registered in a publicly accessible database: Research Registry (U.I.N. researchregistry6872) [2]. This research was approved by the Institutional Research Ethics Board (B.U.N. 143201836187), in accordance with the revised Helsinki Declaration about reporting experiments on human subjects and has been reported in line with the STROCSS criteria [3]. Our Craniofacial Anomalies Database was reviewed for initial diagnosis, gender, associated syndrome, and necessity for velopharyngeal surgery.

The cohort series comprised cases of consecutive non-syndromic cleft lip, alveolus, and palate operated by the same surgeon (MM) or his co-workers/trainees in two tertiary hospitals in Belgium with full records. In cases of missing information, the patients were contacted through e-mail and post. We excluded revisional cases, syndromic including Pierre Robin sequence patients, patients with submucosal clefts, and patients younger than 5 years old.

Secondly, a systematic review of literature was conducted. The PICO framework for quantitative studies was used to develop the search strategy.

This literature review has been reported in line with PRISMA (Preferred Reporting Items for Systematic Reviews and Meta-Analyses) [4]. The study population (P) included cleft palate patients (with or without lip and/or alveolus cleft) who underwent primary palatoplasty (I). The outcome measure in our series was the necessity for a secondary procedure to correct velopharyngeal insufficiency (O). The results of systematic review were compared with the results of our case series (C), which we treated using a standardized cleft protocol.

### 2.2 Literature search strategy and selection criteria

A comprehensive online search was carried out. The included databases were *PubMed, EMBASE, Cochrane Database of Systematic Reviews* and *Science Direct*. No language or timeline restrictions were applied. The following sequence was used for PubMed: (((velopharyngeal insufficiency) OR velopharyngeal incompetence OR velopharyngoplasty) AND cleft palate) OR palatoplasty with ‘cleft palate’ and ‘velopharyngeal insufficiency’ as Medical Subject Heading terms. The sequence was adapted to each database.

The inclusion criteria chosen to select the potential articles were as follows: 1) human patients, 2) patients who underwent primary cleft palate surgery, 3) syndromic and non-syndromic patients including Pierre Robin sequence, 4) only articles in academic publications, 5) timing and technique of the primary surgery must be cited in the article, 6) age of the last speech assessment or follow-up mentioned, 7) number of patients who underwent secondary surgery correction for the VPI mentioned, and 8) articles with level I-IV of evidence (level V articles excluded). There were no restrictions regarding group size.

### 2.3 Eligibility criteria and article selection process

Two independent reviewers assessed article eligibility as follows as follows: the titles and abstracts were scrutinized to remove the non-topic articles. Afterwards, the full text of the potentially relevant studies was retrieved and translated if needed. Lastly, the references of the selected articles were hand searched.

### 2.4 Surgical protocol and palatal closure technique

Our protocol for primary cleft surgery consisted of lip/nose adhesion at 3-4 weeks of age, definitive lip repair according to Millard–Mohler–Asensio and primary.

Millard–McComb rhinoplasty at 4 months of age for unilateral complete clefts and Millard-Mulliken cheilorhinoplasty for bilateral complete clefts. The soft palate was repaired according to Widmaier-Perko or Furlow between 9 and 12 months of age (depending on speech development). Fibrin sealant was used in to eliminate the dead spaces in the lateral pharynx between the oral and nasal layers [5] and as a wound pack over the oral sutures. Quilting sutures for further obliteration of dead spaces [6].

The hard palate was repaired at 4 years of age using pairing of the edges in case of a narrow palatal cleft (usually in incomplete unilateral clefts), and hinge-door flaps in combination with a flip-over flap [7] in the rest of the cases. A single transpositional palatal flap was raised for wider unilateral complete clefts (with or without relaxing incision) and double transpositional palatal flaps in bilateral complete cases. The alveolar repair with iliac bone graft was performed between the age of 8–11 years.

### 2.5 Speech therapy

Our current protocol is based on early speech and language therapy: an immediate postnatal meeting between the therapist, child, and parents is succeeded by regular follow-up of developmental speech and language milestones. Until 2012, speech therapy was only initiated after definitive closure of the palate, around 4.5 years. From 2012 on, early therapy was commenced in cases of compensatory articulation pattern detection, even before palate repair.

When perceptual analysis and acoustic speech recordings confirmed a problem with the velopharyngeal valve, objective measurements were performed, including nasometry, videofluoroscopy or nasopharyngoscopy to assess velopharyngeal closure and nasal airflow rates. Nasometry is one of the most used instrumental evaluations and our first choice. Because the amount of nasal energy depends on the architecture of the nasal and pharyngeal passages and the presence of an intact hard and soft palate, nasometry was performed in our cohort only after definitive palate closure [7].

These methods, in conjunction with clinical speech evaluation were used to determine whether the next step in treatment planning includes surgical intervention and/or speech therapy. The following parameters were investigated by the speech therapist: hypernasality, hyponasality, audible nasal air emission and/or nasal turbulence, consonant production errors and voice disorders [8,9].

The decision which determined which path should the treatment of the velopharyngeal incompetence follow (conservative or surgical) is made in our center at 5 years. The age of 5 years was chosen because children start primary school in Belgium at the age of 6 years. According to the literature children with velopharyngeal sufﬁciency at this age are highly unlikely to develop subsequent VPI [10]. Moreover, cooperation of young children, by nature, differs with age: children under 4 years are harder to cooperate and to allow for the appropriate diagnostic tests in order determine whether any secondary surgical intervention is required [11]. As noted by other authors children of 5 years of age and older, will show good cooperation in a familiar setting [12].

### 2.6 Data extraction

The following parameters were extracted by one author (AT) from the full text of each selected article: first author, year of publication, number of patients, patient gender and age at the time of palatoplasty, syndromic/non-syndromic, type of Veau cleft, study design (randomized controlled trial, prospective, or retrospective), level of evidence (as study quality index) (see Table 1). To evaluate the quality of research (level I-IV), the Level of Evidence scale was used in accord with the 2011 Oxford Center for Evidence-Based Medicine (CEBM) recommendations [13].

**Table 1:** Data extraction discriminated by author.

**Author**	**Year**	**Type of study**	**LOE**	**N**	**n**	**Gender(male)**	**S/NS**	**S**	**NS**	**Age at speech evaluation(years)**	**F–U**	**Primary palate surgery**	**Timing of palatoplasty**
Furlow	1986	retro	III	22	0	NR	NS	0	22	4	3.8	Furlow Z-plasty	10.8
Enemark et al.	1990	prosp	III	57	13	42	NS	0	57	5	21	vomer flap + Push back palatoplasty	24
Gunther et al.	1998	retro	III	52	15	NR	S + NS	NC		3	10	intravelar velopasty	12.1
				24	2	NR						Furlow Z-plasty	11.3
Marrinan et al.	1998	retro	III	72	10	NR	NS	0	72	4	4	Von Langenbeck	8–16
				156	23			0	156	4		Veau-Wardill-Kilner	8–16
Kirschner et al.	1999	retro	III	181	13	NR	NS	0	181	8.5	7.7	modified Furlow Z-plasy	10.1
Becker et al.	2000	retro	III	44	21	17	NR	NR	NR	24	18	Von Langenbeck	7.1
				22	8	8				20		Wardill	8.2
Pulkkinen et al.	2001	retro	III	65	16	35	NS	0	65	8	8	Veau-Wardill-Kilner/Cronin modification	12–24 m
Bicknell et al.	2002	retro	III	114	28	63	NR	NR	NR	6	6	Veau-Wardill-Kilner/von Langenbeck/Furlow Z-plasty + intravelar velopasty	6–18 m
Schnitt et al.	2004	retro	III	22	7	19	NS	0	22	8	16	2-flap pushback palatoplasty	13.6
LaRossa et al.	2004	retro	III	262	17	NR	NS	0	262	8	7	modified Furlow Z-plasy	10.5
Inman et al.	2005	retro	III	124	31	NR	NR	NR	NR	NR	17	Veau-Wardill-Kilner/von Langenbeck	12
Holland et al.	2007	retro	III	41	8	23	NS	0	41	6	15	unipedicled mucoperiosteal flaps and vomer flaps	13
				41	26	25		0	41			modified Von Langenbeck	12
Andrades et al.	2008	retro	III	110	27	66	S + NS	17	93	4.6	NR	Two-flap palatoplasty	12.2
				103	2	59		17	86	3.1		Two-flap palatoplasty + radical intravelar veloplasty	12.6
Khosla et al.	2008	retro	III	140	3	73	S + NS	18	122	4.9	3.5	Furlow modified (acc to Randall)+vomer flaps	12
Phua et al.	2008	retro	III	211	28	108	S + NS	33	178	NR	4.3	Veau/von Langenbeck/Furlow/other	13
Farzaneh et al.	2009	retro	III	34	9	23	NR	NR	NR	28	19	Von Langenbeck	8
				27	4	12				21		Wardill	18
Koh et al.	2009	retro	III	15	4	NR	NR	NR	NR		6.2	Classic 2-flap palatoplasty	11.2
				15	2	NR		NR	NR		4.3	Modified 2-flap palatoplasty (dissection only of the medial border on the noncleft side)	12.2
Sperry	2009	retro	III	256	17	162	NS	0	256	4	4	2-flap palatoplasty	9.3
Sullivan et al.	2009	retro	III	449	67	246	NS	0	449	4	4	2-flap palatoplasty	11.6
Goudy et al.	2011	retro	III	21	3	13	NS(PRS)	0	21	NR	8	3-flap palatoplasty	14.2
				42	10	NR	NS	0	42	NR	NR		12.5
Lohmander et al.	2012	long	III	55	6	41	NS	0	55	19	19	Gothenburg protocol, vomer flap	7.5
Zhao et al.	2012	retro	III	224	67	130	NS	0	224	NR	5	2-flap palatoplasty with classic intravelar velopasty/Sommerlad technique	67.2
Jackson et al.	2013	retro	IV	559	45	307	NS	0	559	5	8.8	modified Furlow Z-plasy	12.3
Mahoney et al.	2013	retro	III	485	50	276	S + NS	NR	NR	NR	10	Furlow/Veau/von Langenbeck/hybrid/other	20.4
Stransky et al.	2013	retro	III	55	11	NR	NS-PRS	0	55	8.9	7.83	modified Furlow Z-plasty	13
				129	16	NR	NS-NPRS	0	129	8.5	7.52		12
Lithovius et al.	2014	retro	III	138	29	61	NR	NR	NR	NR		3-layer palatoplasty + intravelar veloplasty	6–24
Ha et al. .	2015	retro	III	292	56	147	NS	0	292	3	3	Z-plasty/2-flap palatoplasty/intravelar veloplasty/von Langenbeck	11.9
Hosseinabad et al.	2015	retro	III	131	47	76	NR	NR	NR	4	4	Veau-Wardill-Kilner/von Langenbeck	18.49
Follmar et al.	2015	retro obsv	III	183	23	96	S + NS	10	173	NR	5	von Langenbeck/Furlow/2-flap palatoplasty and intravelar veloplasty	10
				18	6	6		1	17				31
Elander et al.	2016	retro	III	94	16	37	S + NS	NR	NR	NR	10	Gothenburg 2 - staged protocol	7.7
Yamaguchi et al.	2016	retro	IV	231	6	104	NS	0	231	3	3	Modified Furlow-plasty + Von Langenbeck/2-flap palatoplasty/straight method	8.3
Yuan et al.	2016	retro obvs	III	177	9	84	S + NS	NR	NR	NR	3.8	von Langenbeck/Furlow/2-flap palatoplasty/one-flap palatoplasty	10.1
Chorney et al.	2017	retro	III	312	16	160	S + NS	27	285	NR	6.49	modified Furlow Z-plasty	9.5
Kappen et al.	2017	retro	III	48	19	35	NS	0	48	NR	21	intravelar veloplasty (Perko)+ Von Langenbeck	7.5
Mann et al.	2017	retro	III	303	20	NR	S + NS	NR	NR	5	7.76	Furlow Z-plasty ± buccal flap	8–12
Moren et al.	2017	retro	III	47	8	NR	NS	0	47	NR	39	Veau-Wardill and the Skoog modification	21
				26	3	NR	NS	0	26		26	As described in the text	18
Klintö et al.	2019	retro	III	10	2	7	NS	0	10	NR	5	Gothenburg protocol	7.2
				8	0	2		0	8			Linköping protocol (Bardach palatoplasty)	19.5
				9	0	8		0	9			Malmö protocol (acc to Sommerlad)	11.3
				10	4	6		0	10			Stockholm protocol (Von Langenbeck/von Langenbeck + Veau-Wardill Kilner)	12.5
				10	0	7		0	10			Umeå protocol (acc to Sommerlad/Gothenburg protocol)	7.5
				10	2	7		0	10			Uppsala-Örebo protocol (acc to Sommerlad)	7.1
Pai et al.	2019	retro	IV	72	30	32	NS	0	72	4	21.3	Bardach palatoplasty	12
N:number of patients		S/NS:syndromic/Non-syndromic patients											
F–U:follow-up		NR:not recorded											
LOE:level of evidence		NC:can not be calculated											
n:number of patients who underwent secondary velopharyngoplasty													

### 2.7 Statistical analysis

Data of studies included for review were analyzed using IBM SPSS Statistics for Microsoft, v22.0 (IBM Corp, Armonk, NY, USA). A limited descriptive statistical analysis was performed. Results were presented as means and percentages. Standard deviation was determined as measure of data dispersion. Student *t*-test was calculated and p-value < 0.05 was considered statistically significant. Chi-squared test (χ2) was carried out. Binomial regression (Wald Chi-Square test) was used to assess the relationship between a binary response variable and other explanatory variables. Pearson correlation coefficient measured strength between the different variables and their relationships.

## 3 Results

### 3.1 Retrospective cohort study

We performed descriptive statistics of our case series comprised of patients primarily diagnosed and operated between May 1990 and August 2019.

The pool of included patients was divided in two categories: patients who underwent a Widmaier-Perko closure of the soft palate (operated between May 1990 and March 1994) and patients who underwent a Furlow palatoplasty (operated between April 1994 and August 2019).

Of the 24 Widmaier-Perko subjects with palatal clefting, two syndromic cases were excluded (Pierre-Robin Sequence and Trisomy 21) leaving 22 cases for analysis:

3 (13.6%) had a Veau I type of cleft, 5 (22.7%) had a Veau II type cleft, 9 (40.9%) had a Veau III type cleft and 5 (22.7%) had a Veau IV type cleft. Two (9%) of these 22 cleft individuals required a pharyngoplasty.

The distribution of secondary pharyngeal surgery was as follows: 0/3 for Veau I patients, 1/5 (20%) for Veau II patients, 0/9 for Veau III patients, and 1/5 (20%) for Veau IV patients. The gender distribution ratio was 1:1.

Of the 179 Furlow subjects with palatal clefting, syndromic cases were excluded as follows: Opitz Syndrome (1), Van der Woude Syndrome (1), Cerebro-costo-mandibular syndrome (1), Stickler Syndrome (1), Pierre Robin Sequence (26), Velocardiofacial syndrome (1), Chromosome 18 Ring (1). A final number of 147 patients were included for analysis: 54 (36.7%) had a Veau I type of cleft, 25 (17%) had a Veau II type cleft, 47 (32%) had a Veau III type cleft and 21 (14.3%) had a Veau IV type cleft.

Two (1.3%) of these 147 cleft individuals required a pharyngoplasty. One additional patient underwent synthetic calcium hydroxyapatite pharyngoplasty injection (Radiesse Voice ®, Merz Aesthetics) into the posterior and lateral wall of the pharynx. The distribution of secondary pharyngeal surgery was as follows: 0/54 for Veau I patients, 1/25 (4%) for Veau II patients, 1/47 (2.1%) for Veau III patients, and 0/21 for Veau IV patients. The gender distribution ratio was 1:1.

From the total number of patients included in our study, 5 patients (2.9%) required secondary pharyngoplasty. However, the difference between Furlow and Widmaier-Perko group proved statistically not significant (p = 0.083).

### 3.2 Literature search

**Fig. 1:**
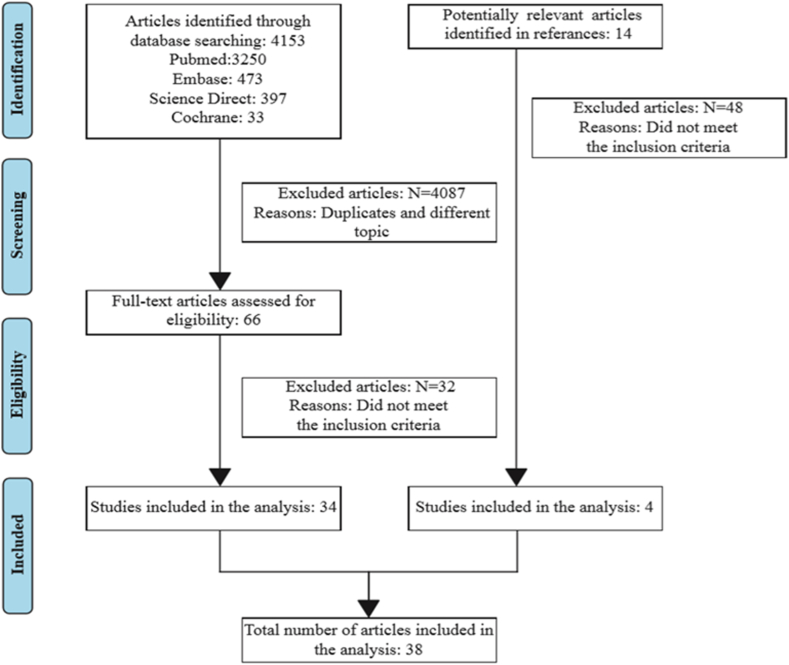
PRISMA flow chart.

The literature search yielded 4153 studies, of which 34 met the inclusion criteria. Another 4 articles were found by searching the references of the included studies. The selection process is depicted in Fig. 1. The final selection included 38 studies.

### 3.3 Methodological quality of the included studies

We analyzed the possible sources of variability or heterogeneity among the included studies. Clinical heterogeneity arose from differences in surgical protocols (one-staged versus two-staged), speech evaluation parameters, timing of outcome measurements and intervention characteristics (different palatoplasty techniques). The methodological heterogeneity arose from the variability in the risk of bias of the included studies.

To handle heterogeneity, we followed the strategies described in the Cochrane Handbook for Systematic Reviews and Interventions [14]. As such the most transparent approach was to present the data without performing a meta-analysis.

### 3.4 Selected studies

One study was prospective of design [15] (2.6%), one was longitudinal [16] (2.6%) and 36 were retrospective (94.7%). The mean age at the time of speech assessment was 9.93 years. From the total 6316 number of patients included in the analysis, 895 (14.2%) underwent secondary velopharyngeal surgery.

The frequency of surgery to correct VPI after one- and two-stage protocols were 13.6% (780/5750) and 24.5% (87/355), respectively. The Chi-square test proved the difference to be statistically significant (χ2 = 35.425, df = 1, p < 0.0001).

The frequency of secondary velopharyngeal surgery in isolated cleft palate patients (Veau I and II types) was 13.6% (94/1425), compared to 15.8% (247/1562) in unilateral (Veau III type) clefts and 13.5% (69/513) in bilateral (Veau IV type) clefts.

Cleft type was a significant factor in the frequency of pharyngoplasty surgery. Pearson r correlation test demonstrated that isolated cleft palate group is strongly associated with pharyngoplasty surgery (Pearson r = 0.876, p < 0.05) compared to cleft lip, palate and alveolus group (Pearson r = 0.685, p < 0.05).

No statistical difference was found between bilateral and unilateral clefts (p > 0.05).

The frequencies of velopharyngeal surgery were 7.2% after a primary Furlow palatoplasty, 17.5% after a 2-flap palatoplasty, 18.6% after a Wardill-Killner palatoplasty, and 35.6% after a Von Langenbeck palatoplasty. None of the included studies referred to the posterior wall augmentation.

Pierre Robin sequence patients had a velopharyngeal surgery frequency of 12.4% compared to 8.6% in the non-Pierre Robin sequence group. The patients operated following a one-stage protocol were divided in three groups according to age at palatoplasty: before 9 months, between 9 and 12 month and after 12 months. The binomial regression determined that age is not significant predictor of incidence of secondary pharyngeal surgery (Wald Chi-Square = 5.797, df = 2, p = 0.055).

Gender was not a significant predictor of the incidence of secondary surgery. The difference in incidence between male and female proved statistically not significant (Wald Chi-Square = 0.417, df = 1, p = 0.518).

Perceptual speech assessment was performed in all included articles. The number of speech therapists who participated in the speech assessment was missing in 8 studies (21%). In 12 (31.6%) of the 38 articles, only one listener had been used for speech evaluation and two or more listeners were used in the rest 18 studies (47.4%). Inter-rater reliability was recorded in 9 articles (23.7%).

The most common speech variable evaluated in the studies was hypernasality followed by nasal air emission and articulation. Consonant production errors, grimace, voice, and intelligibility were also used as variables. Nine studies used hypernasality as only speech parameter. Velopharyngeal function was not scored uniformly across the studies with the same scale. The most common method for assessment used was an interval rating scales with 3 to 10-point scales. A composite speech score for each subject was then calculated by adding the scores for each individual variable.

In 12 articles (28.9%) instrumental analysis complemented the perceptual evaluation: visual (videofluoroscopy, nasoendoscopy, cephalometry) and acoustic measurements (nasometry).

### 3.5 Assessment of bias risk

**Fig. 2:**
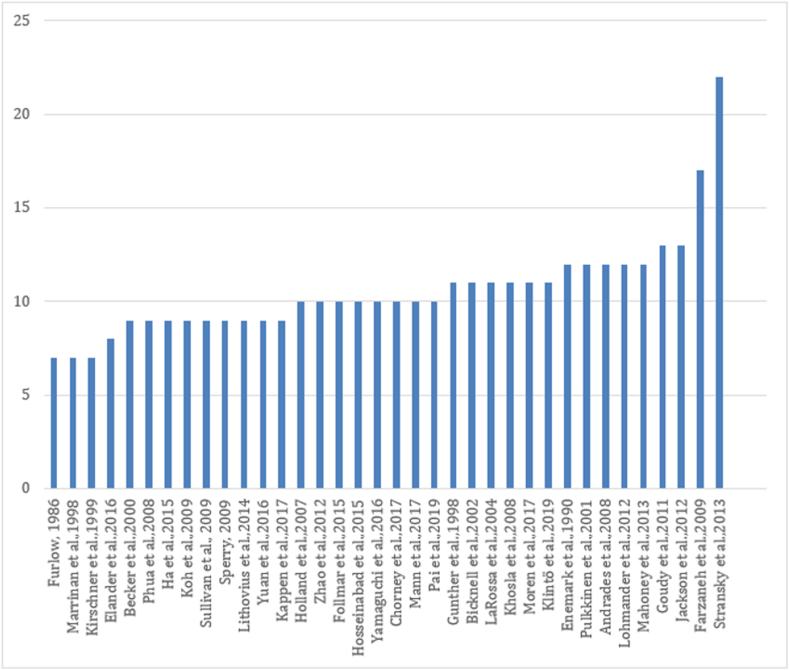
Risk of bias.

The MINORS (Methodological Index for Non-Randomized Studies) tool was used to assess risk of bias in non-randomized study results [17]. The general risks of bias are individually presented in Fig. 2. Bias in non-randomized studies showed considerable variability (mean 10.34, SD 2.86, CI 95%), which contributed to the generally found heterogeneity. To avoid selective reporting, no articles were excluded based on the assessed risk of bias [18]_._

## 4 Discussion

VPI deeply impacts communication and building of social relationships between patients and their family and friends [11]. Thus, achieving intelligible speech is an important outcome measure for primary palatal repair.

The main measure of pharyngeal competence in our series was the necessity for a secondary procedure to correct velopharyngeal insufficiency.

Choosing between a one- and two-stage protocol has been subject of debate in the literature. Some authors suggest the two-staged protocol is associated with poorer speech outcomes [19,20]. In contrast, the cleft center in Gothenburg reported better velopharyngeal function after the two-stage protocol. Lohmander et al. [16] and Morén et al. [21] found no significant difference between the two protocols.

Most studies included in this systematic review adhered to a one-stage protocol. We found that the frequency of secondary velopharyngeal surgery after one-stage protocol is 13.6% compared to 24.5% after a two-staged palatal closure.

The frequency of pharyngoplasty surgery in our patient group following a two-staged protocol was 2.9%. Our decision to perform a secondary pharyngoplasty was based upon the speech pathologist's evaluation as determined by perceptual and instrumental measures. As mentioned earlier the evaluation was performed in preschool period, in accordance with most of the body of literature included in this review. Questions were raised about adenoid involution at a later age and developing hypernasality in cleft palate patients. However, studies show that not all patients use their adenoid pad in attempted velopharyngeal closure [22] and patients who show midline velar-pharyngeal contact against adenoid tissue between the ages of 5 and 7 years are not likely to lose that contact during subsequent years following normal atrophy of the adenoidal pad [23].

Through this literature review, we found what other factors besides stage and timing of palatoplasty may contribute to poorer speech outcome after palatoplasty. Type of clefting proved to be a significant variable in the frequency of pharyngoplasty surgery. Patients with isolated cleft palate have a stronger association with a higher incidence of pharyngoplasty surgery than patients with cleft lip, palate and alveolus which confirms the results of previous studies [[24], [25], [26]].

The highest incidence of secondary velopharyngeal surgery was noted in the Veau III type group (15.8%) but without significant difference compared to the Veau IV group (13.5%).

Gender was considered a dependent variable in determining the frequency of secondary pharyngoplasty in some studies. Bicknell et al. [27] and Hosseinabad et al. [28] reported more severe hypernasality in boys compared to girls. Lithovius et al. [1] found a higher need for subsequent pharyngoplasty in girls than in boys. The results of our systematic review support the hypothesis that gender does not influence VPI frequency.

Many palatal cleft repair techniques have been described in literature, but none has surged as being ideal. Furlow's double opposing Z-plasty proved most successful in achieving palatal lengthening and was associated with the lowest rate of secondary pharyngeal surgery. On the other end of the spectrum, the Von Langenbeck straight-line closure is associated with the highest rate of secondary pharyngoplasty.

Our results corroborate previous studies comparing Pierre Robin sequence and non-Pierre Robin sequence patients. We conclude that there is no significant difference between non-syndromic patients with and without Robin sequence regarding the rate of secondary surgery for velopharyngeal incompetence [29,30].

The role of surgeon's experience was analyzed in four studies [24,[31], [32], [33]] as it is assumed that surgical experience has a learning curve and surgical skills improve over time [32]. Speech outcomes were noted to correlate with surgical experience: the incidence of second surgery to treat postoperative VPI decreased with increasing surgical experience.

This study poses some limitations that need to be addressed. The perceptual speech assessment in the included studies proved inconsistent even though protocols have been developed and universal speech parameters were devised in previous literature [9]. When discussing palatoplasties, some centres use multiple techniques, precluding an accurate analysis. Data completeness for retrospective studies depended upon medical records, so some inaccuracy may occur.

## 5 Conclusion

The literature reported that one-stage palatoplasty is correlated with a lower incidence of secondary pharyngeal surgery compared to a two-stage protocol. Our standardized two-stage protocol proved successful in avoiding secondary velopharyngeal surgery but due to the reduced number of patients included in our study, more research is needed.

## Ethical approval

A retrospective observational study was designed and approved by the Institutional Research Ethics Board of the Universitair Ziekenhuis Brussel-Vrije Universiteit Brussel, Brussels, Belgium (B.U.N. 143201836187).

## Author contribution

Ana Tache, MD, MSc^1-^ study design, data collection, data analysis, writing; Youri Maryn, MSc, PhD^2^ study design, revised the article critically for important intellectual content and approved the final version of the article to be published; Maurice Y. Mommaerts, MD, DMD, PhD, FEBOMS, FICS, FAACS^1^ study design, writing, revised the article critically for important intellectual content and approved the final version of the article to be published.

## Consent

Written informed consent was obtained from the patient for publication of this case report and accompanying images. A copy of the written consent is available for review by the Editor-in-Chief of this journal on request.

## Registration of research studies

Name of the registry: www.researchregistry.com.

Unique Identifying number or registration ID: researchregistry6872.

Hyperlink to your specific registration (must be publicly accessible and will be checked): https://www.researchregistry.com/browse-the-registry#home/.

## Guarantor

Ana Tache.

## Funding

None.

## Declaration of competing interest

No conflict of interest.
